# Book Review: Roma Minority Youth Across Cultural Contexts Taking a Positive Approach to Research, Policy, and Practice

**DOI:** 10.3389/fpsyg.2021.746280

**Published:** 2021-08-31

**Authors:** Snezana Stupar-Rutenfrans, Aaron Johnson

**Affiliations:** Department of Social Sciences, University College Roosevelt, Middelburg, Netherlands

**Keywords:** positive youth development, ethnic minority, Roma, assessment, acculturation

Radosveta Dimitrova, David Sam, and Laura Ferrer-Wreder (Oxford: Oxford University Press), 2021, 264 pages, ISBN: 9780190654061 https://global.oup.com/academic/product/roma-minority-youth-across-cultural-contexts-9780190654061?q=dimitrova&lang=en&cc=us#

This Oxford University Press volume edited by Radosvet Dimitrova, David Sam, and Laura Ferrer-Wreder is concerned with investigating the most stigmatized and marginalized ethnic minority in Europe from a global cultural perspective. This book is about Positive Youth Development (PYD) among Roma ethnic minority youth across cultures by bringing about their shared sociocultural world through participatory and broader collective processes, while at the same time highlighting how the Roma youth are experiencing these social processes. The key insight that runs through this interdisciplinary collection of 13 chapters is the focus on a large and underrepresented Roma ethnic minority group in a strength-based conception of adolescence (i.e., PYD). Positive Youth Development (PYD) scholarship has exploded within the social and health sciences, delineating the processes of optimal well-being, thriving, and success among young people. This book presents contributions of new PYD scholarship globally and in marginalized understudied contexts, and areas of growth in PYD among ethnic minority groups and cultures.

The volume begins with a foreword by one of the founders of PYD, Prof. Richard M. Lerner and comprises three parts as follows. Part A describes a brief historical overview of major socio-demographic, cultural, and contextual characteristics of Roma populations across different settings. Part B presents theories on adaptation focusing on strengths-based and PYD traditional frameworks. Part C presents empirical findings on PYD and well-being of Roma youth in a number of countries of settlement in a culturally sensitive perspective of development. Therefore, the whole volume pays special attention to cultural specifics and universals of the findings to the human and adolescent development in other similarly oppressed minority groups. Policy implications are offered to the readers with suggestions on improving life conditions of the next generation of Roma in a global perspective.

What makes the volume particularly attractive for a global audience within the Cultural Psychology specialty section is its cultural-ecological foundations to the study of human development and ethnic minority Roma youth. The wide range of contributions report qualitative and quantitative empirical findings outside the WEIRD settings and mainstream psychology. Such work offers invaluable support in identifying cultural mechanisms and insights of group differences, similarities, and patterns of development that inform cultural foundations and assumptions on the study of ethnic minority groups. From such standpoints, this book examines psychological and acculturation processes across multiple groups of Roma people who vary in engagement with different cultural-ecological patterns, multiple cultural identities and contexts. Across chapters, the authors reveal the cultural foundations of adaptive patterns among Roma with solid measurements and assessment of core psychological processes (e.g., identity, well-being, thriving, acculturation, family connectedness). Further, the international and multidisciplinary set of contributors addresses the complexities of Roma life in a variety of cultural settings, as to provide a better contextual understanding of their life experiences and settings and promote PYD as a global science.

The original goals have been accomplished: PYD and positive psychology within cultural science are increasingly attracting attention with a focus on being culturally inclusive and responsive to the diverse needs and experiences of young people in a global perspective. Consequently, as that approach has matured, it has also become much more effective in exploring how key developmental processes and person-context interactions can contribute to optimal and successful adaptation. As this volume demonstrates well, the PYD approach is now widely applied and suitable for a conception of culture as dynamic and fluid patterns affecting Roma minority youth. It is thereby developing an attractive vision for a modern psychological science that places cultural interaction, especially socio cultural interaction, center stage.

## Author Contributions

SS-R and AJ: writing and reviewing. Both authors contributed to the article and approved the submitted version.

## Conflict of Interest

The authors declare that the research was conducted in the absence of any commercial or financial relationships that could be construed as a potential conflict of interest.

## Publisher's Note

All claims expressed in this article are solely those of the authors and do not necessarily represent those of their affiliated organizations, or those of the publisher, the editors and the reviewers. Any product that may be evaluated in this article, or claim that may be made by its manufacturer, is not guaranteed or endorsed by the publisher.

